# Exploring quality of care through the eyes of formal and informal caregivers for residents with Huntington's disease: A qualitative descriptive study

**DOI:** 10.1177/18796397251410253

**Published:** 2026-02-17

**Authors:** Joyce CF Heffels, Irma HJ Everink, Raymund AC Roos, Jos MGA Schols, Mayke Oosterloo

**Affiliations:** 1 Department of Health Services Research and Care and Public Health Research Institute (CAPHRI), 168092 Maastricht University, Maastricht, The Netherlands; 2 Huntington's Disease Center Land van Horne, Weert, The Netherlands; 3 168089 Institute of Mental Health and Neuroscience, Maastricht University, Maastricht, The Netherlands; 4 Department of Neurology, 4501 Leiden University Medical Center, Leiden, The Netherlands; 5 Department of Family Medicine and Care and Public Health Research Institute (CAPHRI), Maastricht University, Maastricht, The Netherlands; 6 Department of Neurology, 199236 Maastricht University Medical Center, Maastricht, The Netherlands

**Keywords:** qualitative research, focus groups, Huntington's disease, informal caregiver, formal caregiver, quality of care

## Abstract

**Aim:**

To gain insight into factors contributing to the quality of care in nursing homes specialized in Huntington's disease from the perspectives of formal and informal caregivers.

**Design:**

A qualitative descriptive focus group study.

**Methods:**

Formal and informal caregivers from three specialized nursing homes in the Netherlands were separated into six focus groups. All data was analyzed using reflexive thematic analysis.

**Results:**

From both groups, three main themes were explored: (1) care approaches and principles, (2) attention to specific care domains, and (3) preconditions for delivering care. Formal caregivers highlighted the importance of balancing residents’ autonomy and restrictions, the need to work systematically, and adopting a resident-centered approach. Furthermore, attention to palliative care, nutritional care, and emotional support are needed. Informal caregivers emphasized the need for providing structure, supportive conversations, and the importance of the nursing home environment itself. Attention to the transition phase immediately after admission to the nursing home, as well as guidance on future care planning, is important. There is a need for care teams that consist of knowledgeable, familiar caregivers within a care organization that (im)materially supports its staff.

**Conclusion:**

Maintaining autonomy despite disease-related restrictions, providing structure, applying palliative care principles, and offering emotional support are essential components of quality of care, as emphasized by both formal and informal caregivers. Achieving this requires systematic nursing practices performed by a team of knowledgeable caregivers. Although informal caregivers’ descriptions are more superficial, key elements in them are similar to those of formal caregivers.

## Plain language summary

**What was the aim of this study?:**

This study explored how to provide good care for people with Huntington's disease living in specialized nursing homes. We wanted to understand what both professional caregivers (such as nurses and care staff) and family members consider important for high-quality care.

**How was the study done?:**

We held group discussions with professional caregivers and family members from three Dutch nursing homes that specialize in Huntington's disease. We analyzed what they shared about their experiences and views.

**What did we find?:**

Professional caregivers mentioned the importance of putting the resident at the center of care and working in a systematic way. They also spoke about the need for supporting autonomy while taking into account necessary restrictions. In addition, caregivers highlighted the need for emotional support, and attention to specific areas such as palliative care and nutrition. Family members emphasized the need for daily structure, supportive conversations, and a pleasant nursing home environment. They also said that support is needed right after a resident moves in, and that guidance and planning for the future are crucial. Both groups emphasized the importance of a skilled and familiar care team with sufficient knowledge of the disease.

**What does this mean?:**

Providing good care for people with Huntington's disease requires a combination of supporting independence, offering structure, providing emotional and palliative care, and working as a consistent and knowledgeable team. While professionals and family members bring different perspectives, they largely agreed on what matters most in Huntington's disease care.

## Introduction

Huntington's disease (HD) is a progressive, inheritable neurodegenerative disorder characterized by motor, cognitive, and psychiatric symptoms.^1,2^ Over the course of their disease, patients with HD require specialized care, as their condition leads to complete dependency in daily functioning and eventually death.^1,3,4^ In most cases the progression requires admission to a nursing home in later stages.^5–7^ In the Netherlands, seven specialized nursing homes provide care mainly in the advanced stages of HD, distinguishing themselves from regular nursing homes by tailoring the activities, environment, and behavioral approaches to meet the unique needs of individuals with HD.^8^ However, caring for patients with HD is challenging for healthcare professionals even in a specialized setting. The multifaceted nature of HD symptoms requires a comprehensive and individualized care plan, as well as caregivers who possess knowledge and expertise about the disease. The disease also imposes a large burden on informal caregivers due to its progressive nature, often young age of onset, and complexity of symptoms.

When examining quality of care (QoC) in the nursing home setting, the concept is complex and dynamic, shaped by the perspectives of key stakeholders.^9,10^ It encompasses not only the effectiveness and safety of care, but also the extent to which care meets the needs, preferences, and expectations of residents.^11–14^ Because of their involvement in daily care, both formal and informal caregivers play an essential role in the experience and evaluation of QoC. In this study, QoC is therefore defined as the aspects of care that are most meaningful to those directly involved in daily practice. Moreover, QoC includes elements that can be actively influenced by the nursing home itself.

There has been a notable shift over the last two decades toward frameworks of person-centered and relationship-centered care in nursing homes to improve QoC.^15,16^ Person-centered care prioritizes the resident's voice and applies a more integral approach that looks at the resident's individual preferences, needs, and expectations,^17,18^ while relationship-centered care looks at the relationships between caregivers and residents and acknowledges their impact on QoC.^19,20^ The INDEXQUAL model^21^ integrates these perspectives by conceptualizing QoC in a nursing home setting, underscoring a care triad consisting of the resident, informal caregiver, and formal caregiver. The perspective of each member of this triad is essential for providing person-centered care in a relationship-centered approach.^20,22,23^

Despite the growing recognition of these frameworks, research on QoC in the context of HD remains limited. Currently, methods to assess QoC in nursing homes are available for the general nursing home population but not for patients with HD.^24,25^ Yet, the unique characteristics of HD demand HD-specific attention to QoC.

In this, it is crucial to understand the residents’ perspectives when assessing the experienced QoC in specialized HD nursing homes.^26^ In a recent study,^27^ we gained insight into the perspectives of residents with HD and identified several key QoC domains, including: a) experiences with care and daily living, b) experiences with living environment, autonomy, and well-being, c) the role of formal caregivers, and d) fellow resident interaction. However, this resident-focused view provides only a partial understanding of QoC. Although one study recently investigated the perspectives of formal caregivers,^28^ no studies have systematically examined the perspectives of both formal and informal caregivers within HD-specialized nursing homes, to form an integral view of and comprehensively address care needs and quality perspectives.

This study addresses that gap by exploring perspectives of formal and informal caregivers as it is essential for exploring care models that are both person- and relationship-centered. Therefore, in this study, we aim to gain insight into factors contributing to QoC in Dutch HD-specialized nursing homes from the perspectives of formal and informal caregivers.

## Methods

### Design

This qualitative descriptive study used focus groups with formal and informal caregivers of residents with HD. Focus groups were chosen to facilitate interaction between participants, allowing them to share experiences and build on each other's perspectives.^29^ Six focus groups were conducted with participants from three specialized HD nursing homes in the Netherlands.

### Theoretical framework

Based on our previously conducted review,^25^ eight domains were identified as essential for assessing QoC in early-onset neurodegenerative diseases, including HD. These domains include: emotional support, physical support, social support, care, care content, expertise, communication, and organization of care. A topic list was developed using these eight QoC domains as a framework. All domains were transformed into questions and sub-questions. The emotional support-domain, for instance, led to the following questions on the topic list: a) What do you think of emotional support in the context of QoC? b) What kind of emotional support is needed? c) Is emotional support an important topic in the context of QoC? and d) When, how, and by whom is emotional support needed? The interview questions can be found in Additional file 1.

### Study setting and recruitment

Participants were selected from the following nursing homes specialized in HD care: Atlant (Apeldoorn), Mijzo (Raamsdonksveer), and Land van Horne (Weert). The homes all provide care to at least 25 residents with HD, with a notable concentration of staff with experience and expertise. Each nursing home is further divided into units with individual private rooms and shared living areas. In addition, all three nursing homes are affiliated with the Huntington Knowledge Network Netherlands (HKNN), which ensures compliance with nationally set quality criteria for caring for residents with HD.^8^

Potential participants were identified by the contact person working at each nursing home, who provided them with written information about the study. If caregivers were interested in the study, they were invited to participate in a focus group. In each of the nursing homes, we conducted two focus groups, about 1.5 h each: one with formal caregivers and one with informal caregivers. The focus groups were held on-site.

### Inclusion criteria

Eligible informal caregivers were those aged 18 years or older who were actively involved in the care of their family member. Eligible formal caregivers were healthcare assistants or nurses who were vocationally trained and had been working for over one year for at least 24 h per week within a specialized HD unit.

### Data collection

All focus groups were conducted by JH, who is a trained moderator and healthcare professional specialized in the care of people with HD. The focus groups were audio recorded. Participants were given the opportunity to introduce themselves and ask questions about the study. Several ground rules were explained to create an open and respectful discussion environment. Basic demographics (age, gender) were collected from each participant. Formal caregivers were requested to provide their years of HD-related working experience and hours. For informal caregivers, their relationship to the resident was documented. Both formal and informal caregivers were then asked to share their opinions and understanding of QoC for HD nursing home residents by writing keywords on paper and explaining these keywords. When no new topics were added, participants were asked about each of the eight domains of the topic list and whether they believed the topic was an important QoC item for residents with HD, and how it applied to their own experiences. Participants were encouraged to share concrete examples, elaborate on their experiences, and reflect on contrasting perspectives within the group. In each focus group, participants had the opportunity to add topics if they wished until no new information was shared.

### Data analysis

The analysis process involved several iterative steps, in which we used elements of reflexive thematic analysis.^30^ While the focus group discussions were guided by a literature-informed topic list, the analysis was conducted inductively. Themes were developed independently of the topic guide, based solely on participants’ narratives, as part of an inductive approach of thematic analysis.^30^ This helped us capture the perspectives of the participants without being constrained by predefined categories. All focus groups were transcribed verbatim, anonymized, and subsequently analyzed using Atlas.ti (version 23.1.1.0). During each stage of the analysis, notes of each step were documented to ensure a transparent and systematic process.

The first step of the analysis was to thoroughly read all the transcripts (JH) to become familiar with the content and make initial points of interest. The second step was to search the transcripts for meaningful words and phrases related to the research question (open codes). Two authors (JH and IE) independently coded 25% of one informal and one formal caregiver's transcript. Differences between the two researchers’ codes were discussed until full agreement was met, ensuring inter-coder agreement and enhancing the credibility of the analysis. The remaining transcripts were similarly coded by JH. In the third step of the analysis, the open codes were organized into preliminary themes by one author (JH). This process was independently done based on the transcripts by a research assistant as well. Afterward, the themes were compared, and differences between findings were discussed and adjusted until full agreement was reached. In the fourth step, the preliminary themes were evaluated by JH and the research assistant to ensure they accurately captured the data's essence. If necessary, an iterative process was initiated, involving a secondary coding cycle to ensure a thorough interpretation of the data. Subsequently, recurring themes were categorized into major and minor themes. In the fifth step, each theme was named and given a clear description with a representative title.

### Ethical considerations

The study protocol was approved by the Ethics Committee of Maastricht University Medical Center in The Netherlands (METC 2020-1517). All participants gave their written informed consent.

### Rigor and reflexivity

Reporting of the study and its results was guided by the Standards for Reporting Qualitative Research (SRQR).^31^ All participants were allowed to review and validate the transcripts of their group discussion. Transparency in the data analysis process was maintained by documenting detailed notes at each stage. To promote reflexivity,^32^ several measures were implemented throughout the study. The moderator of the focus groups (JH) has had a previous professional relationship with some participants. It was expected that this familiarity could improve the likelihood of participants sharing experiences openly. Additionally, the multiple steps involved in the data analysis process were discussed within the team, which comprised several researchers with expert knowledge in HD care. This collaborative approach helped to challenge individual interpretations, enhanced reflexivity, and supported a richer understanding of the data.

## Results

### Characteristics of participants

The six focus groups were conducted between March 2022 and February 2023 and each lasted between 70 and 95 min. The three focus groups with formal caregivers (*n* *=* *12)* consisted of only female participants (*n* = *5, n* *=* *4, and n* *=* *3*) with a mean age of 45.5 years (range 24–59 years). The three focus groups with informal caregivers included 10 participants (*n* *=* *4, n* *=* *3, and n* *=* *3)*, of whom five were female. The mean age of informal caregivers was 62.5 years (range 48–74 years). An overview of the characteristics of each group is given in Table 1.

**Table 1.** Background characteristics of participants.

Background characteristics	Participants
***Informal caregivers (*n** *=* *10)***	
Female/Male (*n*)	5 / 5
Age in years (mean, range)	62.5 (48–74)
*Relationship to resident (n)*	
Partner	5
Ex-partner	1
Parent	1
Child	1
Sibling	1
Sister-in-law	1
Time of involvement with resident at their current HD nursing home in months (mean, range)	46.6 (4–108)
***Formal caregivers (*n** *=* *12)***	
Female/Male (*n*)	12 / 0
Age in years (mean, range)	45.5 (24–59)
Experience in working with HD in years (mean, range)	4.9 (1–19)
Working hours per week in HD nursing home (mean, range)	29.2 (24–36)
*Function (n)*	
Baccalaureate-educated registered nurse	4
Vocationally trained registered nurse	6
Certified nurse assistant	2

HD: Huntington's Disease.

### Key themes

An overview of the key themes from the formal and informal caregivers is shown in Table 2. In the formal caregiver group, three main themes emerged: (1) Care approaches and principles, (2) Attention to specific care domains, and (3) Preconditions for delivering care. The informal caregiver group showed the same three main themes, which are discussed in sections (4), (5), and (6), respectively.

**Table 2.** Overview of elements of quality of care of residents with Huntington's disease according to formal and informal caregivers.

Key themes for quality of care from the perspective of formal and informal caregivers		
Main theme	Subthemes	Perspective
Care approaches and principles	Autonomy and restrictions	F
	Resident centered care	F
	Systematic nursing practices	F
	Providing structure	I
	Supportive conversations	I
	HD care domains	I
	Nursing home environment	I
Attention to specific care domains	Palliative care	F
	Nutritional and oral care	F
	Emotional support	F
	Supporting personal care	F
	Social relationships and interactions	F
	Transition phase	I
	Future perspective	I
Preconditions for delivering care	Caregiver characteristics	F + I
	Team characteristics	F + I
	Characteristics of the organization	F + I

HD: Huntington's disease. F: Formal caregiver. I: Informal caregiver. F + I: Formal and informal caregiver.

### Key themes for quality of care from the perspective of formal caregivers (themes 1–3)

#### 1 Care approaches and principles

The first theme, “Care approaches and principles,” includes Autonomy and restrictions (1.1), Resident-centered care (1.2), and Systematic nursing practices (1.3).

##### 1.1 Autonomy and restrictions

Formal caregivers stressed the need to continuously balance residents’ autonomy with the challenges of HD in order to deliver high-quality care. On one hand, they should respect residents’ wishes, preferences, and choices. On the other hand, they need to manage the residents’ limited disease insight, reduced sensory processing, and behavioral challenges.

Not all of a resident's preferences are feasible or safe due to their disease-related limitations and organizational constraints. When necessary, formal caregivers have to guide residents toward safe and suitable choices, considering their capabilities, while still allowing them to make decisions. Setting and defining boundaries, and being consistent in making agreements, is essential, according to participants. Each resident needs to have an individual care plan in which this is described, and all team members are required to adhere to these agreements.

“*Setting boundaries always sounds very negative, but in this case, it is not negative. Not even for the residents themselves. Because it's in their mind that something must happen again. And that can also become very demanding at a certain point. It is also exhausting for the residents themselves* [if we don’t give restrictions].” – formal caregiver A

Formal caregivers highlighted the importance of providing structure in residents’ daily lives. They described not only the importance of the timing and planning of activities but also routines, such as the place the resident often sits at the table.

Reducing stimuli is crucial to prevent overstimulation and certain associating behaviors. To prevent overstimulation, formal caregivers need to personalize the way care is provided, such as having only one person talk during personal care. However, for residents suffering from apathy, stimuli are essential for functioning. This difference shows the need for personalized care.

##### 1.2 Resident-centered care

Participants emphasized placing the resident at the center of care provision, stressing the need to work responsively and to consider each individual's unique needs and wishes. This process often requires flexibility, patience and time, and the ability to adjust quickly if needed. Residents need to feel heard and supported in every aspect of their care. Several formal caregivers highlighted the importance of seeing the person behind the disease despite any cognitive or behavioral changes. They also noted that residents’ needs should be discussed as early in the disease process as possible.

Some formal caregivers mentioned that they are the voice of a resident because they have built a strong relationship with them and learned their non-verbal communication signs. Participants in all focus groups pointed out the value of creating a caring environment that feels comfortable and homelike for residents.

“*I also believe that we are doing well if people eventually start seeing this as their home. Then it's not just a house, but a home.”* – formal caregiver B

##### 1.3 Systematic nursing practices

Structured, systematic methods in providing care and clinical reasoning are essential, according to formal caregivers. Continuously evaluating and adjusting care plans was stated to be of utmost importance due to changes in the situation and patients’ conditions. Formal caregivers work with short-term goals to determine what works and make timely adjustments as needed.

Despite the outward appearance of quietness and calmness in an HD unit, which might suggest simplicity to an outsider, achieving this environment requires numerous adjustments and clinical thinking. Some caregivers mentioned that working in an HD unit is energy-consuming, as formal caregivers need to de-escalate situations from getting out of hand.

“*When a colleague is talking with a resident, especially one who is highly stressed or needs gentle guidance, you shouldn’t interfere. You keep your distance and acknowledge that the colleague is handling the situation. If they need help, they will ask, but it's important not to take over the conversation.”* – formal caregiver C

Thinking beyond standard rules whenever necessary was noted, for example performing personal care tasks at night. Formal caregivers also stressed the need to set aside their personal norms and values, recognizing that those of the residents may change due to disease progression. For instance, previously held standards might conflict with new behaviors, such as walking in public without being well-groomed. Changes such as these lead to evaluations and actions to protect residents from themselves.

#### 2 Attention to specific care domains

The second theme, “Attention to specific care domains,” is subdivided into Palliative care (2.1), Nutritional and oral care (2.2), Emotional support (2.3), Supporting personal care (2.4), and Social relationships and interactions (2.5).

##### 2.1 Palliative care

Formal caregivers emphasized that providing attention to aspects of palliative care and discussing sensitive topics, such as resuscitation and tube feeding, with both the resident and informal caregivers, are fundamental aspects of high-quality care, particularly given the progressive nature of HD. Palliative care should be integrated into regular evaluation moments with the resident and informal caregivers, especially in the case of significant decline in health.

“*Palliative care is important, not necessarily terminal care, but truly palliative. It involves looking at what someone still can do, what those moments of energy are worth … What still brings someone quality of life? For example, do you start another course of antibiotics? Or does someone want to be resuscitated?”* – formal caregiver D

##### 2.2 Nutritional and oral care

Formal caregivers highlighted the importance of attention to eating and drinking since residents with HD require an adequate caloric intake due to their increased metabolic rate. Caregivers’ support during mealtimes is crucial due to swallowing difficulties, often in combination with residents’ limited disease awareness. To reduce the risk of choking, it is important to create a calm, stimulus-free environment, for instance by closing the living room door during meals. Formal caregivers mentioned being constantly aware of textures of food and drinks and the risk of choking. They emphasized the need for qualified personnel to assist all residents with eating and drinking.

“*For example, we have eating areas where people face the wall. They also eat at different times, and sometimes the choice of who you sit with at the table is made deliberately. That is intentional.”* – formal caregiver D

Oral care is deemed essential and in need of attention from formal caregivers. It can sometimes be overlooked, especially in the advanced stages of the disease.

##### 2.3 Emotional support

Formal caregivers emphasized the importance of emotional support, primarily through verbal conversations with residents about several topics. Participants mentioned they are often the first point of contact for residents needing support, and they frequently function as confidants. If urgent emotions arise, such as sudden frustration or an emotional outburst, formal caregivers highlight the need to act immediately and de-escalate the situation instead of waiting for a colleague or informal caregiver. In most cases, offering a listening ear is sufficient, making sure not to judge the choices made by the resident. Being honest about the losses caused by the illness and paying attention to bereavement are also crucial themes to discuss with residents.

“*Just last week, I was with someone who said, ‘I’ve messed everything up,’ and I responded, ‘Huntington's only has losers.’ He said, ‘That's absolutely true.’ By saying that, you acknowledge the reality, which he already knows. He knows his wife can no longer support him. But it's about acknowledging that deep sorrow. That it is what it is* – *being heard, seen, and acknowledged.”* – formal caregiver B

Observing the resident with HD is also crucial, especially when verbal communication becomes impossible due to disease severity. Formal caregivers are the eyes and ears of residents, often able to understand non-verbal gestures and cues and obtaining information from informal caregivers. Caregivers should be patient when talking with residents due to delayed information processing, making communication in some cases difficult but still possible. Formal caregivers also need to discuss the impact that the deterioration and death of fellow residents has on individual residents.

##### 2.4 Supporting personal care

Residents need to be physically supported in their care. If residents resist self-care, formal caregivers respect their wishes, unless it becomes a problem or harms the resident's body. Most formal caregivers addressed the importance of stimulating independence, as long as it does not lead to resistance from the resident. Due to the young age of residents in many cases and the possibility to guide rather than take over personal hygiene tasks, physical assistance is often more important in later stages of the disease.

Formal caregivers deviate from standard care schedules to comply with the wishes and preferences of individual residents. An example is assisting a resident in smoking a cigarette at 2:00 am. Formal caregivers only adjust routines if deviations lead to problems or risks, such as disrupting day and night rhythm.

“*We offer 24/7 care. In regular nursing homes, everyone is somewhat pushed into the routine of being in bed by eleven at the latest and being up by 7:00 am. I think it is also important to respect people with HD if they choose to be awake mainly at night instead of during the day; you should not force them into a different structure. That is also a structure, even if it may not entirely match our perception of a healthy structure.”* – formal caregiver C

##### 2.5 Social relationships and interactions

Formal caregivers need to facilitate contact between residents and their social system, keeping the social network as broad as possible for the resident, for as long as possible. This includes supporting outdoor activities, visiting home, and seeing friends and family. This social support includes physical help with transfers to a wheelchair, support with video calling or the use of smartphones, and providing emotional support when family contact is limited.

Formal caregivers also provide social support to family members, primarily by listening and acting as a safety net. Formal caregivers can answer questions about the care process and the disease, but they should not intervene in family relationships. Conversations with family members often include feelings of guilt about the resident's admission, future concerns for their children, or practical issues. Sometimes formal caregivers refer family members to other disciplines within the multidisciplinary team or HD cafés or partner groups.

Additionally, formal caregivers stressed that dealing with families of residents with HD requires a lot of effort. Communication can be intense due to the history of HD within the family, its impact on the entire family, and the likelihood that other family members may have inherited the gene as well.

#### 3 Preconditions for delivering care

The third theme, “Preconditions for delivering care,” contains Caregiver characteristics (3.1), Team characteristics (3.2), and Characteristics of the organization (3.3).

##### 3.1 Caregiver characteristics

All formal caregivers emphasized the necessity of expertise and competency in the team. They underscored the importance of being able to explore new approaches, such as new ways to guide a resident, and thinking outside the box. These skills are as important as knowing about the disease itself. Staying well-informed, sharing expertise, and maintaining communication within the team were also mentioned. Formal caregivers need to be able to trust their colleagues and provide compassionate care, all to ensure team efficacy.

Furthermore, formal caregivers stressed the importance of being both trained and suited to work with residents with HD. In job interviews, it was suggested that the question “What drives you to work with the target group?” should be asked, in addition to whether someone meets the job profile. Despite limited options for new staff due to labor shortages, it was advised to be critical during job applications.

“*Especially the staff in the unit, that they are competent in dealing with this target group. That they know how to handle aggressive behavior but also stand firm, adhere strictly to agreements, and are not susceptible to manipulation. Staff that can effectively manage this target group because they have the appropriate training.”* – formal caregiver E

##### 3.2 Team characteristics

Participants emphasized the need for familiar faces within the care environment, on one hand for residents to maintain a sense of stability, and on the other hand for the caregivers themselves to build trust within the team. Unfamiliar faces potentially encourage residents to test boundaries.

Formal caregivers highlighted the importance of having experienced colleagues training new team members, providing information and education about HD and its symptoms.

“*We have a very high turnover rate, so we also have many people who do not have much experience with Huntington's, not for many years or months. We still need to train new employees. And then I also wonder: How are they trained? Is enough attention paid to someone's slow information processing?”* – formal caregiver F

Teams should have adequate and supportive team efficacy. Team meetings are essential for communication, building trust among caregivers, and thinking alike in managing behavioral problems. Disagreements on care plans should be discussed in meetings with supervision, and protocols established, for instance, for resident interactions. Detailed reports are crucial, and individualized care plans should be developed collaboratively. Caregivers are expected to follow these plans and report any necessary adjustments. Participants also stressed the importance of multidisciplinary involvement, with close communication and timely interventions to provide care for residents.

##### 3.3 Characteristics of the organization

Formal caregivers acknowledged that organizations have limitations, mostly budget constraints, that can impact care for residents with HD, leading to challenges like limited staffing and fewer activity options. Participants emphasized the need for sufficient budget for education, adequate staffing, and a safe working environment. Caregivers need to be permitted to try out new things, always prioritizing the best interests of the residents. One focus group mentioned the importance of maintaining a balance in the number of extra hours a caregiver is asked to work.

Formal caregivers also pointed out the challenges in the transition from daycare to permanent admission to the nursing home, which makes it (reimbursement-wise) impossible for residents to continue enjoying activities in the daycare and the related social contacts.

“*We have a resident who attended the daycare here for four years and eventually moved in. But now she is no longer allowed to join the daycare* [after her admission]*. She used to attend three times a week, and those were her social contacts. Now that's not allowed anymore because it's a different budget. I think they should really make that transition smoother.”* – formal caregiver A

Additionally, formal caregivers mentioned the possibility of collaborating with other HD organizations in the Netherlands and with other care organizations within the care network, aiming to improve the QoC for individual residents.

The need to deploy more informal caregivers in care tasks in the future, in an attempt to partially compensate for the (future) shortage of healthcare personnel, was highlighted in one focus group particularly. Formal caregivers stated that this might negatively impact the QoC for this target group, as it burdens already overburdened informal caregivers even more.

### Key themes for quality of care from the perspective of informal caregivers (themes 4–6)

#### 4 Care approaches and principles

The first theme according to informal caregivers, “Care approaches and principles,” is divided into Providing structure (4.1), Supportive conversations (4.2), HD care domains (4.3), and Nursing home environment (4.4).

##### 4.1 Providing structure

Informal caregivers highlighted the importance of structure and established routines in residents’ daily lives.

“*Structure is important. Because, just like with my husband, when he wants to put on his pants, first the left leg, then the right one. Don’t do it the other way around. Yes, it's very precise.”* – informal caregiver A

Some informal caregivers noted that establishing structure as effectively as possible is crucial because residents can no longer do that themselves, often leading them to becoming passive. Participants mentioned that residents with strict routines are more active. Several informal caregivers highlighted the impact of unannounced changes in routine and structure, without proper communication, leading to an overload of stimuli.

“*Suddenly someone decided to move the coffee break to seven o’clock. I’ve never seen people so overstimulated. They were yelling, getting really agitated* *…* *They get so many stimuli from that* [change in structure]*. It's unbelievable.”* – informal caregiver B

##### 4.2 Supportive conversations

Informal caregivers stressed the importance of emotional support for residents. Both individual conversations and group conversations with all residents are essential. Participants emphasized that formal caregivers should provide personalized attention and show patience, not only in stand-alone conversations but also throughout the day during care tasks. Formal caregivers should also empathize with residents’ past and present life experiences.

“*My sister also says that she likes it when she can talk to someone … There was a time when a fellow resident pushed or hit her, and* [formal caregivers] *took the time to talk to her about what happened and about her feelings. I think that's very important because I think it's a way for her to feel seen as a person, not just as a patient.”* – informal caregiver C

Some informal caregivers did not feel they needed emotional support for themselves, while others believed that having the opportunity to talk with a formal caregiver who understands the disease is important to maintain good QoC. Care decisions are discussed with both the resident and the informal caregivers, allowing for their input. Informal caregivers value regular care meetings, as well as informal gatherings like the partner groups and HD cafés.

##### 4.3 HD care domains

According to informal caregivers, several specific aspects regarding care provision for residents with HD need attention. Participants mentioned the need for formal caregivers to pay attention to personal care, ensuring residents look well-groomed. One participant noted that not all caregivers have a natural awareness of this, but it is important to at least pay attention to the odor of residents who require bed-based care and prevent them from lying in feces for too long. While some residents don’t prioritize grooming, their informal caregivers may prefer, for example, clothes to be changed if food is spilled on them.

Choreatic movements can make activities of daily living (ADL) challenging, and in some cases, an adjustment in the daily structure of assistance is needed, like the number of caregivers helping a resident at once or adjusting to the resident's pace. Because caregivers support residents, some informal caregivers mentioned a possible increase in a resident's independence after admission, from needing full assistance to only needing guidance.

“*When she first came here, she needed a lot of care because she didn’t do anything herself. Now, so many years later, she actually does everything on her own again. They have managed to get her to take care of herself completely. So yes, they have basically taught her to take care of herself again.”* – informal caregiver D

Encouraging residents to join activities appropriate for their disease stage is needed and can lead to new hobbies, such as baking or painting. Some participants are pleased with the involvement of activity supervisors in organizing these activities. Activities like unit vacations or overnight stays with friends are also appreciated.

Eating requires extra attention because of swallowing issues and the risk of choking, which many informal caregivers find particularly concerning. Participants emphasized the need to calmly assist with meals and, when necessary, adjust the environment, such as eating in a private room to prevent risks. Dinner times should also be adjusted to residents’ preferences.

“*The biggest problem is that she chokes, that's just the way it is. The caregivers can’t change that. However, they can prevent it from happening more or less often by taking things more slowly. The calmer you approach her, and the quieter the environment in which you offer her food or drink, the better it goes.”* – informal caregiver E

Some participants view the possibility of smoking as an important aspect of QoC, as many residents with HD smoke. The opportunity to smoke on the premises will be legally prohibited in the Netherlands within a few years, which will require individual solutions.

It is considered important that the provision and individual customization of aids such as wheelchairs proceed quickly and smoothly. Additionally, there should be a focus on exploring options like electric wheelchairs to maintain residents’ freedom for as long as possible.

##### 4.4 Nursing home environment

All participants stated that the most important aspect of the nursing home environment is the atmosphere, which should feel warm, positive, and homelike. The furnishings of the shared rooms are seen as somewhat sparse, although participants know this is needed to provide a functional use of space and stimuli. There should be a balance between homelike and practical decoration. Private rooms can be personalized, although enough space should remain for care tasks. The nursing home should be in a quiet location with a restaurant nearby and outdoor spaces.

Informal caregivers mentioned that larger units can lead to more incidents, and most of them preferred smaller units. Care needs vary across units due to residents’ disease stages, though some participants noticed that in recent years new admissions are being admitted later in their disease process. One participant highlighted the benefit of knowing fellow residents from previous contact at the daycare.

Most informal caregivers who participated visit their loved ones frequently and feel welcome at any time. Further, they mentioned the low visit rate of family and friends, mostly due to shrinking social networks or the challenging symptoms of the disease, such as aggression. A few residents withhold visitors. Informal caregivers generally don’t see it as the formal caregivers’ role to maintain residents’ social connections.

Although specialized HD nursing homes are usually located at a larger traveling distance than regular nursing homes, the higher QoC makes the distance worthwhile. Many participants think that knowledge and expertise in HD care improves the QoC. Additionally, the availability of age-appropriate activities contributes to satisfaction with the choice of nursing home.

#### 5 Attention to specific care domains

The second theme according to informal caregivers includes Transition phase (5.1) and Future perspective (5.2).

##### 5.1 Transition phase

Informal caregivers stressed the importance of the transition phase when residents move to an HD-specialized nursing home. Providing complete information to informal caregivers during and after admission and offering support during this time is essential. Many participants stated that formal caregivers need to get to know the residents well to understand their wishes, preferences, and routines.

“*Looking back, I think that if I had let her be admitted the first time* [when there was the first option for admission]*, she might have been able to function better. Because at that time, she was still quite well. That way,* [caregivers] *could get to know her earlier in the disease process.”* – informal caregiver F

##### 5.2 Future perspective

Informal caregivers prepare for the future needs of the resident, regardless of their length of stay, and are supported in this by formal caregivers. This includes continuously discussing residents’ life wishes, such as resuscitation and tube feeding. Though limited disease insight can make these conversations difficult, the values of the resident should always remain central.

Participants reported that boundaries, often related to end-of-life wishes, set by residents with HD in the earlier stages of the disease are frequently pushed or reconsidered by the resident as the disease progresses, particularly in the most severe HD stage. Informal caregivers mention the difficulty for them to adapt to these new boundaries, particularly when they conflict with previous choices. Support from formal caregivers is necessary to manage changes in the final disease stage.

Additionally, support from formal caregivers regarding end-of-life wishes is important. This may involve redefining boundaries and denying, adapting, or implementing end-of-life wishes.

“*I would have much preferred if she had said, ‘Well, I have no quality of life anymore, I am looking for another way to step out.’ I wish that for* [resident's name]*. But it's going very differently now than what I think or want for her. Yes, the caregivers do support you in this, and they do show you the other side. That it's very different for the resident.”* – informal caregiver G

Participants highlighted the importance of residents continuing to find positive moments and small joys, even as the disease progresses, to maintain their sense of purpose and a meaningful life. Focusing on simple pleasures like favorite foods or activities is important, especially for residents that require bed-based care, whose worlds have become more limited.

#### 6 Preconditions for delivering care

This theme is divided as follows: Characteristics of formal caregivers (6.1), Team characteristics (6.2), and Characteristics of the organization (6.3).

##### 6.1 Caregiver characteristics

Informal caregivers emphasized their preferences for specific qualities in formal caregivers to ensure high-quality care. Formal caregivers should first of all be friendly, loving, and respectful toward residents, being able to create a pleasant and supportive atmosphere in the unit. Patience and humor were also mentioned as valuable.

“[The care staff] *are so kind to her! Even though she was hitting and was angry, they still treated her very kindly.”* – informal caregiver H

Participants stated that formal caregivers should be involved and compassionate, treating residents as nearly equal. They should be motivated to work with this group. Caregivers should be able to take into account the wishes and preferences of the residents, such as those surrounding mealtimes.

“*It's not just a matter of saying, ‘No, you can’t have* [that food] *anymore* [because of the risk of choking]*, so you just won’t get it, period.’ They really do talk to the resident about it … It is her wish, yes, and she accepts the consequences that might come with it. Of course, that will be documented as well, I understand that, but I’m glad it's possible.”* – informal caregiver D

All participants agreed that formal caregivers must be knowledgeable about HD and praised the current level of expertise. New employees should receive proper training and education before working with residents. Staffing shortages sometimes make this challenging. Experience is crucial, but informal caregivers believe it is more easily gained in a specialized HD nursing home.

##### 6.2 Team characteristics

All participants stressed the importance of formal caregivers being familiar with the residents, as it creates structure and calmness and reduces stimuli. While recognizing it is not always feasible, the goal should be to work with a regular and constant team whenever possible. With new staff, there should be a focus on educating them about HD and providing a training period before they begin.

Each resident has a primary nurse to coordinate their care. Informal caregivers highlighted the need for good communication with this primary nurse to address both resident and caregiver needs. Frequent changes in the primary nurse can disrupt trust as residents and informal caregivers have to get to know someone new all over again.

The team is often multidisciplinary. Some informal caregivers have contact with social workers for help with requests like guardianship or simply offering support. Interactions with the elderly care physician (e.g., physicians working in Dutch nursing homes) depend on their knowledge of HD and their ability to discuss issues such as palliative sedation or end-of-life wishes.

##### 6.3 Characteristics of the organization

Participants stressed the need for enough care staff with sufficient time to spend with residents. There should, for example, be adequate time to help someone with eating or activities, without rushing due to disease symptoms. According to informal caregivers, the organization is responsible for maintaining appropriate staffing levels, ensuring a manageable workload, and making sure staff are properly qualified.

Most informal caregivers believe 24/7 supervision is essential, especially when multiple residents with HD are present, to handle emergencies or incidents. They see it as the organization's responsibility to ensure adequate staffing and make regulations to achieve this. Participants emphasize that the inability of residents to raise alarms is a key reason for constant supervision. Additionally, the absence of formal caregivers during their evening visits increases the wish for 24/7 supervision.

“*She absolutely cannot raise an alarm in any way … She can’t even scratch her nose if a fly is on it, so you quickly end up with the situation of … maybe check on her more often when she's in her apartment.”* – informal caregiver E

The organization should facilitate a consistent number of activities each day, as some participants noted a decline in activities on weekends compared to weekdays. Additionally, caregiver training sessions and team-building activities should be staggered to ensure continuity of care, avoiding situations where all caregivers attend simultaneously, leaving residents with unfamiliar substitutes. Communication could also be enhanced at the unit level, particularly regarding changes that affect other residents, although privacy regulations often limit this. Almost no informal caregivers expressed interest in updates about broader organizational matters.

Informal caregivers also discussed access to the residents’ digital care files. Some appreciated the option, while others were unaware of it. One participant struggled with the personal details in the file, feeling it violated privacy, but still used the information to guide conversations with her loved one.

## Discussion

In this study, we identified key aspects that are crucial for formal and informal caregivers in ensuring meaningful QoC for residents with HD living in specialized nursing homes. Both groups of caregivers highlighted the importance of attention to palliative care and preparing for future perspectives, nutritional care, and offering emotional support. According to both groups, there is a need for a care team that consists of knowledgeable, familiar caregivers within a care organization that supports sufficient staff.

However, the groups had different views on certain topics. To begin, informal caregivers highlighted the importance of the transition period after admission to a nursing home. A study on the needs of informal caregivers in general nursing homes for residents with dementia also highlighted the importance of being aware of the transition phase, and described the needs of informal caregivers to include emotional concerns, knowledge about dementia, understanding the nursing home's rules, and having support from their social environment.^33^ Furthermore, formal caregivers in our study stressed the need for a systematic approach to nursing practices, with a focus on continuous evaluation, resident-centered care, and supportive social relationships. Similar findings were reported by van Walsem et al., who examined the challenges faced by formal caregivers working with residents with advanced HD.^28^ Consistent with our results, they highlight the importance of individual competencies for managing complex care situations and providing good care. Overall, the differences in views between informal and formal caregivers in our study reflect the distinct perspectives of both groups, which have been found in regular nursing homes as well.^34^

In many ways, the topics described in this study are similar to care aspects experienced by general nursing home populations, both from the residents’ perspective^35^ and the formal and informal caregivers’ perspectives.^36,37^ The distinctiveness of HD care lies in the nuances of the results. Furthermore, informal caregivers of residents with HD are not the first to underline the importance of having a “sense of home” for residents. This supports the idea that the domains identified in the context of “sense of home” among mixed resident populations (psychogeriatric, physical, or combined diagnosis)^38^ are equally applicable to the context of QoC for residents with HD. Moreover, several studies involving patients in the early stages of HD,^39–41^ as well as in the general nursing home population,^42^ emphasize the importance of guiding both residents and informal caregivers in coping with ambiguous loss and discussing their future, thereby underscoring the need for comprehensive emotional support for families. In addition, although rehabilitation in HD is primarily described in the context of the period before moving to a nursing home,^43,44^ both formal and informal caregivers reported continuing to focus on promoting independence after admission, highlighting the ongoing relevance of rehabilitation in the more advanced stages of the disease.

General nursing home residents are usually older and may have different needs. Patients with young-onset dementia (YOD) show similarities with those with HD since both groups are relatively young and present unique care challenges, such as managing extensive neuropsychiatric symptoms and requiring a different palliative approach.^45^ In a study investigating the quality of life (QoL) of nursing home residents with YOD,^46^ the authors identified supportive conversations with caregivers and encouraging social interactions with family members or fellow residents as key factors in improving social relationships and reducing negative emotions to ultimately improve QoL.^46^ Although QoL and QoC are distinct concepts, both studies highlight the importance of meaningful connections and emotional well-being. The parallels between these findings suggest that such strategies recommended to improve QoL for residents with YOD may also be effective in enhancing QoC for residents with HD. Thus, insights from other target groups could be used to meet the specific needs of HD patients.

Finally, in our previous study^27^ regarding QoC perspectives of residents with HD, they indicated that interactions with fellow residents was a very important part of QoC. However, this topic was not mentioned in the current study. This discrepancy may be explained by the tendency of informal caregivers to focus primarily on their loved ones rather than on group dynamics. Although formal caregivers may have observed interactions between residents, this topic was not discussed during our focus groups. This contrasts with a previous study, where formal caregivers identified verbal and physical aggression towards residents and caregivers as a challenge in HD care.^28^ As an important QoC topic, it is worthwhile for caregivers to recognize it.

### Strengths and limitations of the work

This study is the first one of its kind to provide insights from (in)formal caregivers of residents with HD on domains they regard as crucial regarding the quality of nursing home care. The inclusion of both formal and informal caregivers from three large HD nursing homes gives a broad perspective on experienced QoC for residents with HD. We captured several insights that have not been documented before.

A limitation of our study is that the selection of informal caregivers may have been biased, as it mainly involved caregivers who often visit their loved ones. This is a common limitation in studies that recruit informal caregivers. However, we believe we included the most knowledgeable participants, which added to the richness of our data.

Another limitation is that we did not compare the QoC for individuals with HD in specialized nursing homes with those in regular nursing homes. While most individuals with HD in the Netherlands are admitted to specialized facilities, a small number reside in their regional nursing homes. In contrast, the availability of specialized HD nursing homes is limited in many countries, largely due to differences in the organization and funding of long-term care facilities.^47^ These differences affect not only the structure of regular nursing homes but also the availability of specialized care for specific groups, such as individuals with HD. As a result, the external validity of our findings may be limited, as the QoC experiences reported in this study may not be fully generalizable to settings outside the Netherlands.

### Recommendations for further research

Currently, there is no validated method to assess care quality from the perspectives of residents with HD living in specialized nursing homes or their formal and informal caregivers. Future studies should focus on developing such a method, using insights from this study as well as our previous study on the residents’ perspectives as a basis.

### Implications for policy and practice

It is recommended that formal caregivers and policymakers use the identified domains to enhance person-centered care for patients with HD. Communication about the significance of these domains is essential to ensure they receive the necessary attention in practice and policy, ultimately improving care for these patients.

Education and support should help formal caregivers adopt a systematic nursing approach. Insights from informal caregivers can also be valuable, shaping care practices and policies to better support families of residents with HD. Recognizing the importance of interactions between residents as part of QoC is essential for caregivers and should be addressed in future care initiatives.

## Conclusion

In conclusion, this qualitative study highlights several aspects for improving the QoC for residents with HD from the perspectives of formal and informal caregivers. Formal caregivers emphasized palliative and oral care, balancing residents’ autonomy with restrictions, working systematically, and adopting a resident-centered approach. Informal caregivers stressed the importance of providing structure, supportive conversations, guidance on future care planning, attention to the physical environment (e.g., atmosphere), and social support. Both groups stressed the importance of the care organization in enabling high-quality care by ensuring education, cohesive teams, and sufficient staffing.

These findings are foundational for HD care and the future development of a tailored assessment tool to measure QoC from the perspectives of residents, formal caregivers, and informal caregivers. We recommend further study to create a population-specific measurement method that captures the unique needs of this patient group.

## Supplemental Material

sj-docx-1-hun-10.1177_18796397251410253 - Supplemental material for Exploring quality of care through the eyes of formal and informal caregivers for residents with Huntington's disease: A qualitative descriptive study Supplemental material, sj-docx-1-hun-10.1177_18796397251410253 for Exploring quality of care through the eyes of formal and informal caregivers for residents with Huntington's disease: A qualitative descriptive study by Joyce CF Heffels, Irma HJ Everink, Raymund AC Roos, Jos MGA Schols and Mayke Oosterloo in Journal of Huntington's Disease
