# Supplementary material for: Exploring quality of care through the eyes of formal and informal caregivers for residents with Huntington's disease: A qualitative descriptive study
Source: J Huntingtons Dis. 2026 Feb 17;15(3):439–52. doi: 10.1177/18796397251410253 (PMC13396418; doi:10.1177/18796397251410253)
Supplement: sj-docx-1-hun-10.1177_18796397251410253 - Supplemental material for Exploring quality of care through the eyes of formal and informal caregivers for residents with Huntington's disease: A qualitative descriptive study [file sj-docx-1-hun-10.1177_18796397251410253.docx]

**Additional file 1 Topic list with focus group questions**

HD: Huntington’s disease

**Quality of care**

- What does good quality of care, specifically for residents with HD, mean to you?
- In your opinion, what should quality of care be about?
- Which domains or aspects do you believe are part of the concept of ‘quality of care’?
- What should a unit for residents with HD offer to provide good care?

**Emotional support**

- What kind of emotional support is needed?
- When, how, and by whom is emotional support needed?
- What do you think about emotional support in the context of quality of care?
- What do you think falls under the domain of ‘emotional support’?
- Is emotional support an important topic in the context of quality of care?
- What do you consider important in the quality of care when it comes to emotional support?
- What should an organization or care professional do?
- What needs to be provided to offer emotional support?

**Physical support**

- What kind of physical support is needed?
- When, how, and by whom is physical support needed?
- What do you think about physical support in the context of quality of care?
- What do you think falls under the domain of ‘physical support’?
- Is physical support an important topic in the context of quality of care?
- What do you consider important in the quality of care when it comes to physical support?
- What should an organization or care professional do?
- What needs to be provided to offer physical support?

**Social support**

- What kind of social support is needed?
- When, how, and by whom is social support needed?
- What do you think about social support in the context of quality of care?
- What do you think falls under the domain of ‘social support’?
- Is social support an important topic in the context of quality of care?
- What do you consider important in the quality of care when it comes to social support?
- What should an organization or care professional do?
- What needs to be provided to offer social support?

**Care (content)**

- What kind of care (content) is needed?
- Which components of care are needed?
- When, how, and by whom should care (content) be provided/is care (content) required?
- What do you think about care (content) in the context of quality of care?
- What do you think falls under the domain of ‘care (content)’?
- Is ‘care (content)’ an important topic in the context of quality of care?
- What do you consider important in the quality of care when it comes to care (content)?
- What should an organization or care professional do?
- What needs to be provided to offer care (content)?

**Expertise**

- What kind of expertise is needed?
- When, how, and by whom is expertise needed?
- What do you think about expertise in the context of quality of care?
- What do you think falls under the domain of ‘expertise’?
- Is expertise an important topic in the context of quality of care?
- What do you consider important in the quality of care when it comes to expertise?
- What should an organization or care professional do?
- What needs to be provided to gain expertise?

**Communication**

- What kind of communication is needed?
- When, how, and by whom is communication needed?
- What do you think about communication in the context of quality of care?
- What do you think falls under the domain of ‘communication’?
- Is communication an important topic in the context of quality of care?
- What do you consider important in the quality of care when it comes to communication?
- What should an organization or care professional do?
- What needs to be provided to support communication?

**Organization of care**

- What kind of organization of care is needed?
- When, how, and by whom is organization of care needed?
- What do you think about the organization of care in the context of quality of care?
- What do you think falls under the domain of ‘organization of care’?
- Is organization of care an important topic in the context of quality of care?
- What do you consider important in the quality of care when it comes to organization of care?
- What should an organization or care professional do?
- What needs to be provided to gain a good organization of care?

**Additional questions**

- What do you think are the specific characteristics or unique aspects of a Huntington unit?
- In what ways does caring for people with HD require a different approach compared to other units?
- From your perspective, what are the main differences between a regular somatic or psychogeriatric unit and a Huntington unit?
- Thinking about everything we’ve discussed today, do you feel there are any important topics we haven’t touched on yet?
- Is there anything else you would like to add or share about your experiences?
